# The expression of Toll-like receptors and development of severe sepsis in patients with acute myeloid leukemias after induction chemotherapy

**DOI:** 10.1007/s12032-014-0319-7

**Published:** 2014-11-22

**Authors:** Justyna Rybka, Aleksandra Butrym, Tomasz Wróbel, Bożena Jaźwiec, Ewa Stefanko, Olga Dobrzyńska, Rafał Poręba, Kazimierz Kuliczkowski

**Affiliations:** 1 Department of Hematology, Blood Neoplasms and Bone Marrow Transplantation, Wroclaw Medical University, ul. Pasteura 4, 50-367 Wrocław, Poland; 2 Department of Internal Medicine, Wroclaw Medical University, Wrocław, Poland

**Keywords:** Acute leukemia, Sepsis, Bacterial infection, Toll-like receptors

## Abstract

Toll-like receptors play an important role in the host defense against microorganisms. Sepsis remains a common cause of mortality in patients with acute myeloid leukemia (AML) treated with intensive induction chemotherapy. The expression of TLRs and their association with the development of sepsis in patients with acute myeloid leukemia remains unclear. The aim of this study was to investigate the associations between expression of TLR2, TLR4 and TLR9 and occurrence of sepsis in patients treated with intensive induction chemotherapy for AML. A total of 103 patients with newly diagnosed AML were evaluated. Bone marrow samples were taken before induction therapy. Using quantitative reverse transcriptase PCR, the mRNA expression of genes TLR2, TLR4 and TLR9 was measured. Neutropenic fever occurred in 98 patients. We identified 20 episodes of severe sepsis (20 %). In patients with neutropenic fever, the mRNA expression of TLR2 and TLR4 was significant higher in septic patients than in patients without sepsis symptoms (ΔCt TLR2 0.93 ± 0.82 vs 0.78 ± 0.85 and ΔCt TLR4 0.38 ± 0.29 vs 0.34 ± 0.25). Moreover, we observed that expression of TLR2 and TLR4 was significantly higher in patients with AML and bacterial infection in comparison with group with separate fungal infection (ΔCt TLR2 1.15 ± 1.06 vs 0.66 ± 0.51 and ΔCt TLR4 0.45 ± 0.38 vs 0.21 ± 0.19). Our results suggest that TLRs could be an independent factor for the development of sepsis in patients with acute myeloid leukemias after intensive induction chemotherapy. This observation should be validated by larger study.

## Introduction

Toll-like receptors (TLRs) are a family of transmembrane receptors which play an important role in the host defense against microorganisms. TLRs are mainly expressed in human immune-related cells such as monocytes, neutrophils, macrophages, dendritic cells, T cells, B cells and NK cells [1]. They initiate inflammatory response including the production of cytokines, chemokines and some adhesion molecules [2]. About eleven human TLRs have been identified up to now and each of them participates in a specific intracellular signaling pathway. TLRs 1, 2, 4, 5 and 6 are typical for bacterial products, TLRs 3, 7 and 8 are characteristic for viral infection and TLR-9 is associated with bacterial and viral inflammatory response [3].

Sepsis is a complex clinical syndrome which is connected with activation and dysfunction of the immune system. Many features of the immunopathology of sepsis are still unclear. To date, the outcome of sepsis and sepsis shock is poor despite the development of antibiotics and other supportive care therapies [4].

Toll-like receptors play a key role in the mediation of systemic responses to pathogens during sepsis. Armstrong et al. [5] showed that the expression of TLR-2 and TLR-4 on monocytes in septic patients is higher than in healthy individuals.

Sepsis remains a common cause of mortality in patients with acute myeloid leukemia treated with intensive induction chemotherapy. The expression of TLRs and their association with the development of sepsis in patients with acute myeloid leukemia remains unspecified.

The aim of our study was to investigate the possible associations between expression of TLR2, TLR4 and TLR9 and occurrence of sepsis in patients treated with intensive induction chemotherapy for AML.

## Materials and methods

A total of 103 patients with newly diagnosed acute myeloid leukemia (AML) were examined (47 females and 56 males). The median age of patients was 51 years (range 18–85). The diagnosis was performed according to the WHO criteria for AML. There were 57 patients (55 %) with acute myeloid leukemias (minimally differentiated, without maturation and with maturation) and 46 patients (45 %) with acute myelomonocytic and monoblastic leukemia. All patients were treated with induction chemotherapy including anthracyclines and arabinoside cytosine. Prophylactic oral chinolones were used in all patients. A total of 17 patients (17 %) had favorable cytogenetic/molecular risk, 52 (50 %) patients had intermediate cytogenetic/molecular risk and 34 patients (33 %) had poor cytogenetic/molecular risk.

The healthy control group included 20 age-matched individuals (9 females and 11 males). Bone marrow samples were taken before induction therapy. Samples were collected after informed consent from patients and control group. Using quantitative reverse transcriptase PCR, the mRNA expression of genes TLR2, TLR4 and TLR9 was measured. To establish the expression of TLRs transcripts in CD34+ cells, mRNAs were isolated with Trizol, and cDNAs were prepared with Moloney murine leukemia virus reverse transcriptase. The PCR was performed using ampli-Taq DNA polymerase with denaturation, annealing and elongation. PCR products were separated in agarose gel. The relative quantitation was indicated by cycle threshold (Ct) values. The Ct value of the target genes was normalized (ΔCt) to the Ct value of the GUS gene of the samples.

The results were statistically analyzed using ‘STATISTICA 8.0’. Statistical analysis was performed by means of Mann–Whitney *U* test, and *p* < 0.05 indicated a significant difference. To determine the independent factors of sepsis occurrence, multivariate logistic regression analysis was executed. The analysis involved expression of TLR2, TLR4 and TLR9, cytogenetic risk, age, gender of patients and type of leukemia. *p* < 0.05 were accepted as statistically significant.

Clinical characteristics of patients are summarized in Table 1.

**Table 1:** Clinical data of patients with AML

103 Patients	
Gender	47 F/56 M
Median age	51 (range 18–85)
Diagnosis	AML with minimally differentiated—6
	AML without maturation—22
	AML with maturation—29
	AML myelomonocytic—34
	AML monoblastic—12
Cytogenetic/molecular risk	Favorable risk—17
	Intermediate risk—52
	Poor risk—34
Neutropenic fever	98 patients
Sepsis	20 patients
Infectious agent	62 patients
	Bacterial—45 patients
	Fungal—15 patients
	Viral—2 patients
Sources of infection	Respiratory system—30 patients
	Abdomen—20 patients
	Urinary tract—10 patients
	Skin—2 patients

## Results

Neutropenic fever occurred in 98 patients (95 %). In 62 patients, (60 %) infectious agent was found. In 36 patients, the etiology of neutropenic fever was not determined. We identified 20 episodes of severe sepsis (20 %). A total of 10 patients with symptoms of severe sepsis died from infection. All patients with symptoms of sepsis after induction chemotherapy had poor cytogenetic/molecular risk. Acute myelomonocytic and monoblastic leukemia was diagnosed in 15 patients with sepsis. Gram-negative bacteria were more commonly found in patients with sepsis and included: *Escherichia coli* in 4 patients, *Klebsiella pneumoniae* in 6 patients and *Pseudomonas aeruginosa* in 5 patients. *Enterococcus faecalis* was detected in 5 patients with sepsis. TLR2 and TLR4 mRNA expression was higher in patients with neutropenic fever than in the group with asymptomatic neutropenia after chemotherapy, although the difference was not statistically significant. Among the patients with neutropenic fever, the mRNA expression of TLR2 and TLR4 was significantly higher in septic patients than in patients without sepsis symptoms. Moreover, we observed that expression of TLR2 and TLR4 was significantly higher in patients with AML and bacterial infection in comparison with the group with isolated fungal infection. In comparison with control group, TLR2 and TLR4 mRNA expression was higher in AML patients than in healthy individuals although there was no statistically significant difference (ΔCt TLR2 0.9 ± 0.85 vs 0.82 ± 0.87 and ΔCt TLR4 0.33 ± 0.23 vs 0.29 ± 0.32).

The results are shown in Table 2.

**Table 2:** Correlation between mRNA expression of TLRs and sepsis in AML patients

	Sepsis n = 20	No sepsis n = 83	*p*
ΔCt TLR2	0.93 ± 0.82	0.78 ± 0.85	<0.01
ΔCt TLR4	0.38 ± 0.29	0.34 ± 0.25	<0.01
ΔCt TLR9	0.002 ± 0.001	0.004 ± 0.003	ns

	Bacterial infection n = 42	Fungal infection n = 15	*p*
ΔCt TLR2	1.15 ± 1.06	0.66 ± 0.51	<0.01
ΔCt TLR4	0.45 ± 0.38	0.21 ± 0.19	<0.01
ΔCt TLR9	0.002 ± 0.001	0.003 ± 0.002	ns

*n* number of patients, *ns* not significant

Multivariate logistics regression model revealed that type of leukemia was an independent factor for sepsis occurrence. Patients with myelomonocytic and monoblastic leukemia had higher risk of manifestation of sepsis than other patients. Patients with response after induction chemotherapy (complete remission and partial remission) had significantly lower risk of sepsis occurrence than patients with no response after treatment.

The results are shown in Table 3.

**Table 3:** Multivariate logistics regression analysis results for sepsis occurrence in AML patients

N = 103	Const BO	TLR2	TLR4	TLR9	Age	Sex	Risk	Response	Type of leukemia
	−0.163	−0.249	−0.625	−0.469	0.000	0.029	−0.546	−1.429	1.415
Standard deviation	0.909	0.400	1.091	165.143	0.016	0.501	0.555	0.510	0.542
t(94)	−0.179	−0.623	−0.573	−0.003	0.004	0.058	−0.984	−2.803	2.609
*p*	0.858	0.535	0.568	0.998	0.997	0.954	0.328	0.006	0.011
−95 %CL	−1.968	−1.043	−2.791	−328.365	−0.031	−0.965	−1.648	−2.441	0.338
+95 %CL	1.642	0.545	1.540	327.426	0.031	1.023	0.556	−0.417	2.492
Chi-square	0.032	0.388	0.329	0.000	0.000	0.003	0.968	7.859	6.804
*p*	0.858	0.533	0.566	0.998	0.997	0.954	0.325	0.005	0.009
OR	0.850	0.779	0.535	0.625	1.000	1.029	0.579	0.240	4.116
−95 %CL	0.140	0.352	0.061	0.000	0.969	0.381	0.192	0.087	1.402
+95 %CL	5.166	1.725	4.665		1.032	2.781	1.744	0.659	12.081
OR		0.324	0.307	0.992	1.004	1.029	0.579	0.240	4.116
−95 %CL		0.009	0.005	0.005	0.125	0.381	0.192	0.087	1.402
+95 %CL		11.760	18.387	206.610	8.056	2.781	1.744	0.659	12.081

*CL* confidence limit, *OR* odds ratio

## Discussion

Toll-like receptors play an important role in host defenses and participate in some of pathological processes including sepsis [6]. The expression of TLRs occurs both on the cell surface and on the intracellulary. TLR2 and TLR4 are expressed on the cell surface, and TLR9 works in intracellular space [6, 7]. TLR2 recognizes some of ligands located on a variety of microorganisms such as bacteria, fungi, viruses and parasites. TLR4, in association with co-receptor CD14, identifies lipopolysaccharide (LPS). TLR9 plays a strategic role in the identification of microbial nucleic acids [8]. Recognition of pathogens ligands by TLRs leads to the activation of some signaling pathways and the secretion of proinflammatory cytokines, such as tumor necrosis factor alpha (TNF-α), interleukin-1 (IL-1) and interleukin-8 (IL-8) [8].

Sepsis is a systemic inflammatory response to a microbial infection and remains one of the leading causes of death. The outcome of sepsis and sepsis shock is poor despite the development of new antibiotics and supportive care therapies. Many molecular mechanisms of sepsis are unclear, and it is essential to understand some immunological processes associated with the development of sepsis [4, 9]. In septic patients, TLRs play a significant role in inflammatory response. The TLRs recognize some specific ligands of pathogens and initiate immune response due to TLR-dependent and TLR-independent pathways [10]. Over the recent years, many studies analyzed the role TLRs in sepsis and sepsis shock [8, 11]. Tsujimoto et al. [3] and Armstrong et al. [5] showed that expression of TLRs on monocytes from septic patients was significantly up-regulated in comparison with healthy individuals. Renshaw et al. [12] found that TLRs expression decreased with age. Patients with AML who are newly diagnosed or relapsed and who are receiving cytotoxic chemotherapy are predisposed to sepsis due to bacterial or fungal infections. The explanation of some mechanisms of sepsis could be useful in risk stratification of development of sepsis or sepsis shock in patients with AML during intensive chemotherapy [13]. The exact role of TLRs in the development of sepsis in AML patients is unknown.

The aim of our study was to analyze the expression of TLR2, TLR4 and TLR9 in patients with symptoms of sepsis after intensive induction chemotherapy for AML. We detected mRNA expression of TLR2, TLR4 and TLR9 in 103 patients with AML before the beginning of treatment. Severe sepsis occured in 20 patients after cytostatic therapy. We demonstrate that the mRNA expression of TLR2 and TLR4 before induction chemotherapy was significantly higher in septic patients than in patients without sepsis symptoms. From among 20 patients with sepsis, there were 15 patients with myelomonocytic and monoblastic acute leukemia. It is interesting that in multivariate analysis type of leukemia was an independent factor for sepsis occurrence, and patients with myelomonocytic and monoblastic leukemia had higher risk of the manifestation of sepsis than other patients. It was impossible to compare our observations with other results because there is no data on the role of TLRs in the development of sepsis in AML patients treated with intensive chemotherapy. Perhaps the high expression of TLRs may initiate the systemic response to pathogens during sepsis and promote proliferation and expansion of immune system cells. The high expression of TLRs in patients with symptoms of sepsis could be connected with an increase in the production of inflammatory cytokines and chemokines. It remains unclear what determines such completely different response during sepsis in different patients. Some studies suggest that the outcome of sepsis depends on genetic influence [14, 15]. Arbour et al. [16] showed that patients with polymorphisms in TLR-4 could be hyporesponsive to endotoxin. Septic patients with TLR-4 polymorphism had increased risk of bacterial infection [17]. A better understanding of TLRs biology may disclose new therapeutic approaches for sepsis.

In conclusion, our results suggest that TLRs could become potential biological markers for the development of sepsis in patients with acute myeloid leukemias after intensive induction chemotherapy. However, this observation should be validated by a larger study.
